# Recurrent Subdural Hematoma Revealing Undiagnosed Mild Hemophilia A and Factor XIII Deficiency in an Elderly Patient

**DOI:** 10.7759/cureus.92502

**Published:** 2025-09-16

**Authors:** Shunsuke Yamanishi, Young Ju Kim, Noriaki Ashida, Akiko Hashimoto, Hirofumi Iwahashi, Yoichi Uozumi, Kohkichi Hosoda, Takashi Sasayama, Masamitsu Nishihara

**Affiliations:** 1 Department of Neurosurgery, Kobe University Graduate School of Medicine, Kobe, JPN; 2 Department of Neurosurgery, Kobe City Nishi-Kobe Medical Center, Kobe, JPN; 3 Department of Immunology and Hematology, Kobe City Nishi-Kobe Medical Center, Kobe, JPN; 4 Department of Neurosurgery, Myodani Hospital, Kobe, JPN

**Keywords:** coagulation factor viii, coagulation factor xiii, cranioplasty, hemophilia a, recurrent acute subdural hematoma

## Abstract

Recurrent acute subdural hematoma (ASDH) is uncommon and typically associated with trauma, vascular malformations, or coagulopathies. While hemophilia is usually diagnosed in childhood, mild forms may remain undetected until adulthood, potentially presenting with unexpected perioperative bleeding.

We report the case of a 75-year-old male who sustained a traumatic head injury resulting in ASDH along the falx cerebri and left frontotemporal region. Initial conservative management was followed by neurological deterioration associated with worsening cerebral edema, requiring emergent craniotomy. Postoperative rebleeding occurred twice, necessitating a total of three craniotomies. A coagulation workup after the second reoperation revealed decreased factor VIII ([FVIII] 22%) and factor XIII ([FXIII] 53%) activity. Although FVIII activity had not been checked initially, it remained consistently low during follow-up. The FVIII inhibitor test was negative, and a cross-mixing test supported the diagnosis of congenital hemophilia A. Genetic testing was not performed due to cost and patient preference. After initiating FVIII and FXIII replacement therapy, cranioplasty was performed without further bleeding. The patient recovered with minimal neurological deficits and remains functionally independent.

This case underscores the need to consider undiagnosed congenital hemophilia in elderly patients with recurrent ASDH or unexplained postoperative rebleeding. Early, targeted coagulation studies, including FVIII and FXIII assays, are critical for diagnosis. Our findings suggest that even mild FXIII deficiency may exacerbate bleeding in hemophilia A, highlighting the limitations of routine coagulation tests. Adequate perioperative replacement of FVIII and FXIII can support safe neurosurgical outcomes.

## Introduction

Acute subdural hematoma (ASDH) is a common neurosurgical emergency, most frequently caused by head trauma. Recurrent ASDH following surgical evacuation is relatively rare and is typically associated with vascular abnormalities, coagulopathies, or inadequate intraoperative hemostasis. Among hereditary bleeding disorders, hemophilia A (factor VIII [FVIII] deficiency) is one of the most prevalent. While severe cases are usually diagnosed in childhood, mild hemophilia A may remain asymptomatic and undetected until adulthood, especially in elderly individuals without a prior history of abnormal bleeding [1,2].

Factor XIII (FXIII) deficiency is a rare but important cause of intracranial hemorrhage, often missed by routine coagulation screening. Previous reports have described its role in spontaneous or traumatic intracranial hemorrhage, primarily in pediatric cases [3,4]. In contrast, the coexistence of mild FVIII deficiency and FXIII deficiency is exceptionally rare and has not been previously reported in elderly patients presenting with recurrent ASDH. Such dual coagulopathy may increase the risk of bleeding even in the absence of overt preoperative signs.

This report aims to present a rare case of recurrent ASDH in a 75-year-old man, ultimately found to have previously undiagnosed mild congenital hemophilia A accompanied by concurrent FXIII deficiency. To our knowledge, this is the first reported case of an elderly patient with recurrent ASDH due to this combination, in whom successful cranioplasty was performed under combined FVIII and FXIII replacement therapy.

This case highlights the importance of considering underlying congenital bleeding disorders in elderly patients with unexplained postoperative hemorrhage and offers insight into appropriate diagnostic and perioperative management strategies to avoid repeated surgical interventions.

## Case presentation

A 75-year-old man with no known history of bleeding disorders visited our outpatient clinic after sustaining a mild head injury from a fall. Although his maternal grandfather had a history of stroke, there was no family history suggestive of hemophilia A. He had not started any new medications and had no comorbidities known to affect coagulation. While he had a prior history of hepatitis C virus infection, his liver function tests were within normal limits.

**Figure 1:**
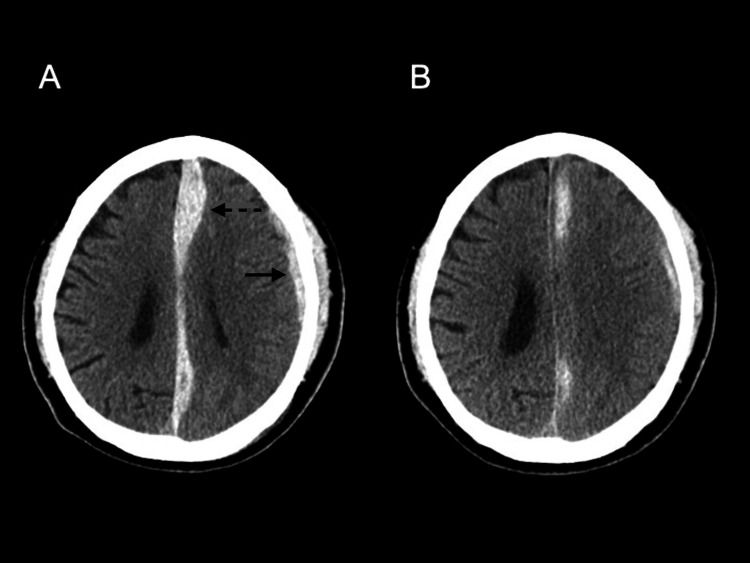
Initial head CT. (A) Acute subdural hematoma located along the falx cerebri (dotted arrow) and in the left frontotemporal region (solid arrow). (B) Increased cerebral edema with mass effect.

Initial non-contrast head CT revealed an ASDH along the falx cerebri and in the left frontotemporal region (Figure 1A), prompting hospital admission for observation. Neurological examination was unremarkable, and conservative management was initiated. On admission, routine laboratory tests showed the following coagulation parameters: platelet count 22.6 × 10⁴/μL, prothrombin time-international normalized ratio (PT-INR) 1.1, activated partial thromboplastin time (APTT) 40.1 seconds, fibrinogen 376 mg/dL, and D-dimer 0.65 μg/mL. Two weeks later, the patient developed decreased consciousness, aphasia, and right hemiparesis. Repeat CT showed increased cerebral edema with mass effect (Figure 1B), necessitating emergency craniotomy.

**Figure 2:**
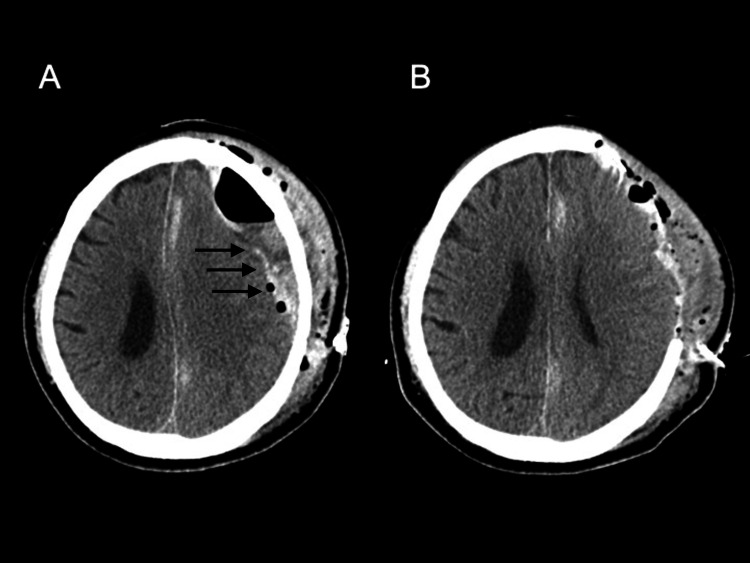
Head CT following hematoma evacuation. (A) Rebleeding (solid arrows) observed after the first craniotomy for hematoma evacuation. (B) Second craniotomy with external decompression performed due to recurrent bleeding.

Despite meticulous intraoperative hemostasis, postoperative CT within 48 hours demonstrated rebleeding at the surgical site. A second craniotomy with external decompression was performed (Figures 2A, 2B), followed by a third craniotomy for further recurrence. During this period, routine coagulation tests remained largely unremarkable, except for a mildly prolonged APTT of 45.7 seconds. No bleeding tendency was suspected at that time.

Given the repeated postoperative hemorrhages, an extended coagulation workup was conducted. This revealed reduced FVIII activity at 22% (reference: 62-145%) and FXIII activity at 53% (reference: >70%), consistent with mild congenital hemophilia A and concurrent FXIII deficiency. The FVIII inhibitor test was negative, and a cross-mixing study confirmed the diagnosis of congenital hemophilia A. Von Willebrand factor levels were within the normal range (157.1%; reference: 50-200%), factor IX activity was 129% (reference: 74-149%), and lupus anticoagulant was negative. Genetic testing was not performed due to high cost and the patient’s decision not to pursue it. Factor XI and XII levels, which primarily affect APTT, were not assessed, as their deficiencies are exceedingly rare and were considered clinically unlikely in this case.

Two months after the initial surgery, cranioplasty was planned and performed under rigorous coagulation management. Perioperative replacement therapy included recombinant FVIII (efraloctocog alfa, 40 IU/kg on the day of surgery and postoperative day 1) and FXIII concentrate (10 IU/kg daily for five consecutive days starting on the day of surgery). FVIII activity was maintained above 80% preoperatively and through postoperative day 2 and between 60% and 80% on postoperative day 3 (Table 1). No postoperative bleeding complications occurred.

**Table 1:** Timeline of clinical events and coagulation test results following head trauma and neurosurgical interventions. APTT, activated partial thromboplastin time; ASDH, acute subdural hematoma; FVIII, factor VIII; FXIII, factor XIII; POD, postoperative day

Day (Post-Injury)	Clinical Event	APTT (seconds) (Ref: 25-40)	FVIII (%) (Ref: 62-145)	FXIII (%) (Ref: >70)	Notes
Day 0	Head trauma and admission	40.1	-	-	ASDH identified on initial CT; managed conservatively
Day 14	1st craniotomy	37.2	-	-	Worsening cerebral edema prompted emergent surgery
Day 15	2nd craniotomy	38.3	-	-	Hematoma evacuation and external decompression
Day 16	3rd craniotomy	45.7	-	-	-
Day 55	Diagnosis of hemophilia A and FXIII deficiency	45.7	22	53	-
Day 74	Cranioplasty under factor replacement	-	22	-	Recombinant FVIII administered ①
Day 74	POD 0	33.7	99	59	Recombinant FXIII administered ①
Day 75	POD 1	36.5	150	93	Recombinant FVIII administered ②
Day 76	POD 2	33.9	89	-	Recombinant FXIII administered ②
Day 77	POD 3	33.9	67	92	Recombinant FXIII administered ③
Day 78	POD 4	-	-	-	Recombinant FXIII administered ④
Day 79	POD 5	-	-	-	Recombinant FXIII administered ⑤
Day 80	POD 6	36.4	28	116	FVIII decreased postoperatively
Day 95	POD 21	36	28	86	FVIII remained low; FXIII remained stable, suggesting transient or acquired deficiency
Day 108	Discharge	-	-	-	-

Shortly after the cranioplasty, during the same hospitalization, his FVIII activity decreased to 28% and remained persistently low until discharge, further supporting the diagnosis of mild congenital hemophilia A. FXIII activity was also monitored and remained stable above 70% until discharge. The sustained response without continued supplementation suggests that the FXIII deficiency was likely acquired and transient rather than congenital.

**Figure 3:**
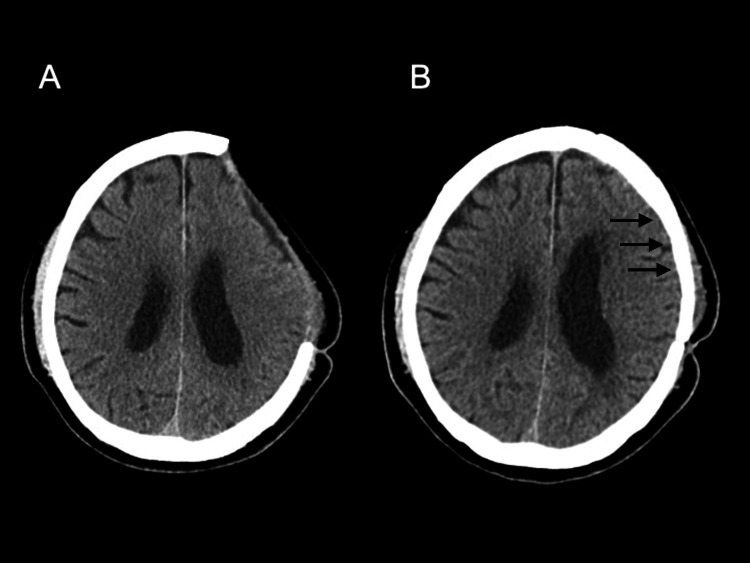
Perioperative head CT for cranioplasty. (A) Preoperative head CT obtained prior to cranioplasty. (B) No postoperative hemorrhagic complications observed following cranioplasty (solid arrows).

The patient was discharged with mild right lower limb weakness but without further hemorrhagic events (Figures 3A, 3B). At follow-up, his neurological symptoms gradually improved, and he is now living independently with minimal functional impairment.

## Discussion

Recurrent bleeding following craniotomy in patients with undiagnosed congenital hemophilia is rare but has been reported in both mild and severe cases [2,5]. This case highlights the importance of considering occult coagulation disorders in elderly patients who present with unexplained postoperative hemorrhage. In the differential diagnosis of unexplained postoperative bleeding, other rare coagulopathies such as FIX (hemophilia B), FXI and FXII deficiencies, antiphospholipid antibody syndrome, and systemic lupus erythematosus should also be considered. In our case, FIX activity and lupus anticoagulant were within the normal range, and FXI/FXII levels were not assessed because of their extreme rarity and the absence of supporting clinical features.

According to the 2020 World Federation of Hemophilia (WFH) Guidelines, hemophilia A is categorized into three severity levels based on FVIII activity: severe (<1%), moderate (1-5%), and mild (5-<40%) [1]. Severe cases often present with spontaneous bleeding into joints or muscles, even without trauma. Moderate cases may show occasional spontaneous bleeding and excessive bleeding after minor trauma or surgical procedures. In mild hemophilia, spontaneous bleeding is uncommon, but patients are still at risk of severe bleeding in the setting of major trauma or surgery. FVIII plays a central role in the intrinsic pathway of coagulation by acting as a cofactor for FIX to activate factor X, ultimately leading to thrombin generation and fibrin formation [6]. Even mild FVIII deficiency can impair thrombin burst and lead to insufficient clot formation, especially when combined with other coagulation defects such as FXIII deficiency. In our patient, FVIII activity was 22%, consistent with mild hemophilia A. Despite this, he developed hematoma expansion and experienced multiple episodes of postoperative rebleeding following only minor head trauma. This unexpected bleeding severity is likely attributable to the coexistence of mild FXIII deficiency.

FXIII, one of the transglutaminases, plays a crucial role in the final phase of the coagulation cascade by cross-linking fibrin monomers to stabilize the clot [7,8]. FXIII deficiency can result in delayed bleeding, wound healing complications, and increased risk of rebleeding, even when initial hemostasis appears adequate. Although intracranial hemorrhage associated with FXIII deficiency has been rarely reported, it remains a plausible and underrecognized cause of persistent surgical site bleeding [3,4,8-11].

Routine coagulation screening, including PT and APTT, may fail to detect mild hemophilia or FXIII deficiency [12]. In our case, PT was normal and APTT only mildly prolonged (40-45 seconds), which did not initially raise suspicion for an underlying bleeding disorder. This underscores the limitations of conventional coagulation tests and highlights the need to perform targeted factor assays when postoperative bleeding is disproportionate to the surgical insult or when rebleeding occurs despite adequate hemostasis. To our knowledge, this is the first reported case of an elderly patient with recurrent ASDH caused by previously undiagnosed mild hemophilia A complicated by FXIII deficiency. Diagnosis in such cases depends on clinical suspicion, particularly in patients without prior bleeding history. Early recognition and targeted replacement therapy are critical to avoid repeated surgical interventions and improve clinical outcomes.

The WFH guidelines recommend maintaining FVIII levels at 80-100% prior to major surgery, 60-80% for the first one to three postoperative days, and 40-60% for the following four to six days [1]. In this case, we administered recombinant FVIII (efraloctocog alfa), which has an extended half-life of approximately 19 hours, around 1.5 times longer than conventional FVIII products, without alteration of specific activity [13]. Our perioperative management closely adhered to these guidelines, resulting in successful hemostasis during and after cranioplasty.

Although FXIII deficiency is rare in patients with hemophilia A, its presence may exacerbate bleeding by impairing fibrin clot cross-linking and long-term stability [14]. In our patient, low FXIII activity likely contributed to persistent oozing and multiple rebleeding episodes. FXIII replacement therapy, typically administered at 10-26 IU/kg, should be considered in similar cases to support clot stabilization and minimize the risk of postoperative hemorrhage [15]. Beckman et al. reported that FXIII cotreatment with hemostatic agents accelerated FXIIIa formation, increased the generation and amount of fibrin α-chain crosslinked species, accelerated α2-antiplasmin crosslinking, and increased clot weight in hemophilia A plasma and whole blood [16]. These findings support the concept that combination therapy with recombinant FVIII and FXIII concentrate may improve hemostatic efficacy in surgical settings.

One of the limitations of this case is the absence of genetic testing for FVIII gene mutations, which would have confirmed the diagnosis of congenital hemophilia A and potentially clarified the inheritance pattern. However, genetic analysis was not pursued due to financial constraints and patient preference.

## Conclusions

This case highlights the importance of considering undiagnosed congenital hemophilia in elderly patients presenting with recurrent ASDH or unexplained postoperative rebleeding. Early, comprehensive coagulation studies, including measurements of FVIII and FXIII activity, are essential to avoid unnecessary repeated surgical interventions. Our report is unique in demonstrating that even mild FXIII deficiency can contribute to persistent bleeding in the context of hemophilia A. This emphasizes the limitations of conventional coagulation tests and underscores the need to perform targeted factor assays when postoperative bleeding is disproportionate to the surgical insult or when rebleeding occurs despite adequate hemostasis. Strict perioperative coagulation management - maintaining FVIII activity above 80% and supplementing FXIII when indicated - can support safe neurosurgical procedures and improve clinical outcomes.

## References

[1] Srivastava A, Santagostino E, Dougall A (2020). WFH Guidelines for the Management of Hemophilia, 3rd edition. Haemophilia.

[2] Ono H, Sase T, Takasuna H, Tanaka Y (2017). Mild hemophilia A presaged by recurrent postoperative hemorrhagic complications in an elderly patient. Surg Neurol Int.

[3] Poombal F, Fayyaz HN, Saeed H, Adhikari S (2024). Factor XIII deficiency leading to a life-threatening intracranial hemorrhage in a young female: a case report. Ann Med Surg (Lond).

[4] Farah RA, Al Danaf JZ, Chahinian RA (2014). Spontaneous epidural hematoma in a child with inherited factor XIII deficiency. J Pediatr Hematol Oncol.

[5] Chopra P, Singh M, Singh A, Masi A, Yurkofsky J, Zaita B, Kaur G (2023). Perioperative management of spontaneous intracranial hemorrhage in a patient with hemophilia A in a resource limited country. Cureus.

[6] Tanaka KA, Terada R, Butt AL, Mazzeffi MA, McNeil JS (2023). Factor VIII: a dynamic modulator of hemostasis and thrombosis in trauma. Anesth Analg.

[7] Vrettou CS, Stavrinou LC, Halikias S, Kyriakopoulou M, Kollias S, Stranjalis G, Koutsoukou A (2010). Factor XIII deficiency as a potential cause of supratentorial haemorrhage after posterior fossa surgery. Acta Neurochir (Wien).

[8] Kawano H, Yamamoto D, Uchihashi Y (2013). Severe inhibitor-negative acquired factor XIII/13 deficiency with aggressive subdural haemorrhage. Blood Coagul Fibrinolysis.

[9] Gerlach R, Raabe A, Zimmermann M, Siegemund A, Seifert V (2000). Factor XIII deficiency and postoperative hemorrhage after neurosurgical procedures. Surg Neurol.

[10] Gerlach R, Tölle F, Raabe A, Zimmermann M, Siegemund A, Seifert V (2002). Increased risk for postoperative hemorrhage after intracranial surgery in patients with decreased factor XIII activity: implications of a prospective study. Stroke.

[11] Perez DL, Diamond EL, Castro CM, Diaz A, Buonanno F, Nogueira RG, Sheth K (2011). Factor XIII deficiency related recurrent spontaneous intracerebral hemorrhage: a case and literature review. Clin Neurol Neurosurg.

[12] Sahud MA (2000). Factor VIII inhibitors. Laboratory diagnosis of inhibitors. Semin Thromb Hemost.

[13] George LA, Camire RM (2015). Profile of efraloctocog alfa and its potential in the treatment of hemophilia A. J Blood Med.

[14] Dorgalaleh A, Tabibian S, Hosseini MS (2016). Diagnosis of factor XIII deficiency. Hematology.

[15] Naderi M, Dorgalaleh A, Alizadeh S, Tabibian S, Hosseini S, Shamsizadeh M, Bamedi T (2014). Clinical manifestations and management of life-threatening bleeding in the largest group of patients with severe factor XIII deficiency. Int J Hematol.

[16] Beckman JD, Holle LA, Wolberg AS (2018). Factor XIII cotreatment with hemostatic agents in hemophilia A increases fibrin α-chain crosslinking. J Thromb Haemost.

